# The need to be seen: social approval and evaluative fears as psychological pathways to FOMO

**DOI:** 10.3389/fpsyg.2026.1840811

**Published:** 2026-06-16

**Authors:** Sinem Evin Akbay, Ali Ammar Kurt, Bilge Bakir Aygar, Şaziye Yaman

**Affiliations:** 1 Department of Psychological Counseling and Guidance, Mersin University, Mersin, Türkiye; 2 Liberal Arts Department, American University of the Middle East, Egaila, Kuwait

**Keywords:** emerging adulthood, fear of missing out, fear of negative evaluation, need for social approval, perceived social competence

## Abstract

**Introduction:**

Fear of missing out (FOMO) has been increasingly linked to social-evaluative concerns and approval-seeking motivations, yet the psychological mechanisms underlying these associations remain insufficiently modeled. The present study examined the mediating role of need for social approval in the relationships between fear of negative evaluation (FNE), perceived social competence, and FOMO among emerging adults.

**Methods:**

Data were collected from 365 university students (aged 18–23) in Türkiye using a cross-sectional design. Two bootstrapped mediation models were tested.

**Results:**

In Model 1, need for social approval fully mediated the statistical association between FNE and FOMO, with no significant direct effect remaining. In Model 2, perceived social competence showed a positive direct association with FOMO alongside a small indirect effect in the opposite direction through reduced approval needs.

**Discussion:**

These findings suggest that FOMO among emerging adults is statistically accounted for largely by approval-based motivational processes rather than evaluative fears alone. Interventions targeting approval-seeking tendencies may be more effective than approaches focused solely on reducing digital exposure. Implications for social-cognitive conceptualizations of FOMO are discussed.

## Introduction

Fear of missing out (FOMO) is defined as a pervasive apprehension that others might be having rewarding experiences from which one is absent (Przybylski et al., 2013). Early conceptualizations positioned FOMO primarily within a needs-based framework, linking it to the frustration of fundamental relatedness needs and fears of social exclusion (Przybylski et al., 2013). Subsequent research has broadened this picture considerably: FOMO has been associated with negative affect, psychological distress, and problematic technology use (Elhai et al., 2021), suggesting that FOMO warrants examination within a broader emotional and motivational framework that encompasses both exclusion-based and evaluative processes—associations with distress and problematic technology use being equally consistent with either account. Studies with university students have further demonstrated that FOMO is significantly related to social media addiction and is associated with psychological variables such as cognitive emotion regulation and life satisfaction (Bakır Aygar et al., 2025). Collectively, these findings indicate that FOMO is not a phenomenon limited to behavioral outcomes but reflects individuals’ broader psychological functioning.

The digital environment has fundamentally transformed the nature of social interaction, with particular consequences for how individuals experience social evaluation. Social media platforms render social feedback publicly visible and quantitatively measurable through metrics such as likes, comments, and follower counts, inviting continuous social comparison (Vogel et al., 2014). In such contexts, social concern shifts: the issue is not merely missing an event, but missing opportunities to be seen, validated, and socially accepted. This transformation suggests that evaluative processes—including concerns about being seen, validated, and socially accepted—may represent an important and underexplored dimension of FOMO experiences alongside exclusion-based concerns (Elhai et al., 2021; Wegmann et al., 2017). Despite this, a substantial portion of the FOMO literature continues to explain the phenomenon predominantly through exclusion-based motivation, leaving evaluative processes relatively underexplored.

Fear of negative evaluation (FNE) is a central construct within this evaluative framework. FNE refers to a persistent apprehension about being judged unfavorably by others (Watson and Friend, 1969). Occupying a central position in the social anxiety literature, FNE leads individuals to become hypervigilant in social situations and to develop heightened self-monitoring of their own performance (Leary, 1983). In digital environments, where social feedback is immediate, public, and permanent, this evaluative sensitivity may be particularly activated (Elhai et al., 2021). Consistent with this, FNE has been shown to positively predict social media addiction (Piko et al., 2024) and to play a mediating role in social media behaviors more broadly (Feng et al., 2025). Direct associations between FNE and FOMO have also been reported, though the number of studies addressing this link specifically remains limited (Alabri, 2022; Wolniewicz et al., 2018). Reer et al. (2019) found that social anxiety, of which FNE is a core component, was associated with FOMO in online gaming contexts, and Dempsey et al. (2019) demonstrated that FNE was positively associated with passive social media use—a behavior closely linked to FOMO. The theoretical basis for expecting a positive FNE–FOMO association is therefore supported by both theory and emerging empirical evidence. It is important to note, however, that FNE is not solely a marker of evaluative threat: FNE is also closely tied to rejection sensitivity and social anxiety more broadly (Watson and Friend, 1969; Leary, 1983), constructs that link evaluative concern to fears of social exclusion. Sociometer theory (Leary and Baumeister, 2000) proposes that self-esteem functions as a monitor of social inclusion, such that fear of negative evaluation may itself reflect an underlying sensitivity to rejection rather than a purely evaluative process. The distinction between evaluation-based and exclusion-based accounts of FOMO is therefore not absolute, and the present study’s focus on approval-seeking as a candidate pathway should be understood as complementing rather than replacing exclusion-based frameworks. The psychological mechanism through which the FNE–FOMO association operates, however, has not yet been sufficiently clarified. Within social anxiety theory, FNE represents the cognitive core of social evaluative concern—the apprehension that others are forming unfavorable impressions (Leary, 1983). Social comparison theory further suggests that individuals high in FNE engage in more frequent upward social comparison, as they are hypervigilant to cues indicating that others are performing or appearing better than themselves (Festinger, 1954). In digitally mediated environments, where such cues are continuously and publicly available, this evaluative sensitivity is likely to translate into heightened FOMO.

The need for social approval offers a theoretically grounded candidate for this mediating mechanism. Need for social approval refers to the motivation to obtain others’ acceptance and positive feedback as a means of maintaining self-worth (Karaşar and Öğülmüş, 2016). Theoretical frameworks proposing that self-worth is contingently tied to external validation suggest that individuals who ground their sense of self in social approval become particularly sensitive to evaluative threat (Crocker and Wolfe, 2001; Crocker et al., 2003). The contingencies of self-worth model (Crocker and Wolfe, 2001) proposes that individuals who ground their self-worth in social approval become acutely sensitive to evaluative threat, responding to it with intensified approval-seeking behavior. Self-determination theory further provides a motivational account of how this process connects to FOMO: when relatedness needs are contingently and externally satisfied, individuals become chronically vigilant about missing opportunities for social connection and recognition (Deci and Ryan, 2000)—a motivational state that maps directly onto FOMO experiences. Consistent with this, Przybylski et al. (2013), who originally conceptualized FOMO within an SDT framework, demonstrated that relatedness need frustration was a key predictor of FOMO. From this perspective, FNE may heighten the need for social approval, which in turn may intensify FOMO: evaluative threat may fuel fear of missing out through the pursuit of external validation. Supporting this sequence, Yoshizawa (2020) found that rejection-avoidance-based approval motivation was positively related to FNE, and Zhu et al. (2024) reported that evaluative anxiety and approval needs were among the strongest co-occurring constructs in a network analysis of social anxiety and FOMO among university students. Furthermore, a positive association between need for social approval and FOMO has been demonstrated directly (Liang, 2017), consistent with evidence that FOMO is related to the pursuit of social acceptance and popularity (Tanrıkulu and Mouratidis, 2023; Li and Liu, 2023).

Perceived social competence occupies a more complex and theoretically distinct position in this model. Perceived social competence refers to individuals’ self-assessments of their effectiveness and proficiency in social interactions (Smith and Betz, 2000). Higher perceived social competence is typically associated with lower social anxiety and reduced evaluative concern (Mürtezaoğlu and Çikrıkci, 2022). This protective function would suggest that socially competent individuals, by depending less on external validation, might experience lower FOMO through a reduced need for social approval. At the same time, however, the relationship between social competence and FOMO may not be unidirectional. Individuals who perceive themselves as socially competent tend to engage more actively in social environments and to maintain broader social networks (Blackwell et al., 2017). This greater social exposure may increase sensitivity to social comparison cues and missed opportunities, thereby elevating FOMO despite lower evaluative anxiety. This dual dynamic—a protective indirect pathway through reduced approval needs and a risk-conferring direct pathway through increased social engagement—is consistent with the “rich-get-richer” hypothesis (Desjarlais and Willoughby, 2010), which posits that socially skilled individuals leverage digital environments to extend already active social lives. Significant associations between perceived social competence and FOMO have been reported in prior research (Tunc-Aksan and Akbay, 2019; Deniz, 2021), supporting the relevance of this construct to FOMO experiences. Social learning theory (Bandura, 1977) provides the theoretical basis for expecting a negative association between perceived social competence and need for social approval: individuals with stronger perceived social competence possess greater internal resources for self-regulation and are consequently less dependent on external validation to maintain self-worth. At the same time, the rich-get-richer hypothesis (Desjarlais and Willoughby, 2010) predicts a positive direct association between social competence and FOMO, positing that socially skilled individuals extend their already active social lives into digital environments, thereby increasing their exposure to social comparison cues and missed opportunities.

The emerging adulthood period renders these dynamics particularly salient. Emerging adulthood, spanning roughly the late teens through the mid-twenties, is characterized by identity exploration, heightened peer orientation, and the central importance of social acceptance (Arnett, 2000). University students navigate intense social evaluation in both offline and online environments simultaneously. Approval-based motivations and evaluative concerns are therefore likely to be especially pronounced in this developmental period, making the proposed pathways particularly visible and meaningful.

The present study makes several contributions to the existing literature. First, although FNE and need for social approval have each been independently associated with FOMO, no prior study has directly modeled need for social approval as a mediating mechanism in the FNE–FOMO relationship. The present study addresses this gap by testing a theoretically grounded indirect pathway through which evaluative fears may translate into FOMO experiences. Second, the role of perceived social competence in FOMO has received limited empirical attention, and no prior study has examined whether it operates through the same approval-based mechanism in a qualitatively different direction. By testing both constructs within a shared mediational framework, the present study provides a more differentiated account of the social-cognitive antecedents of FOMO than has previously been available. Third, the study was conducted with a Turkish emerging adult sample, a context in which approval-seeking motivations may carry particular salience given the collectivist cultural orientation that characterizes Turkish society (Hofstede, 2001). Together, these features position the present study as a theoretically driven and culturally situated extension of the FOMO literature.

**Figure 1:**
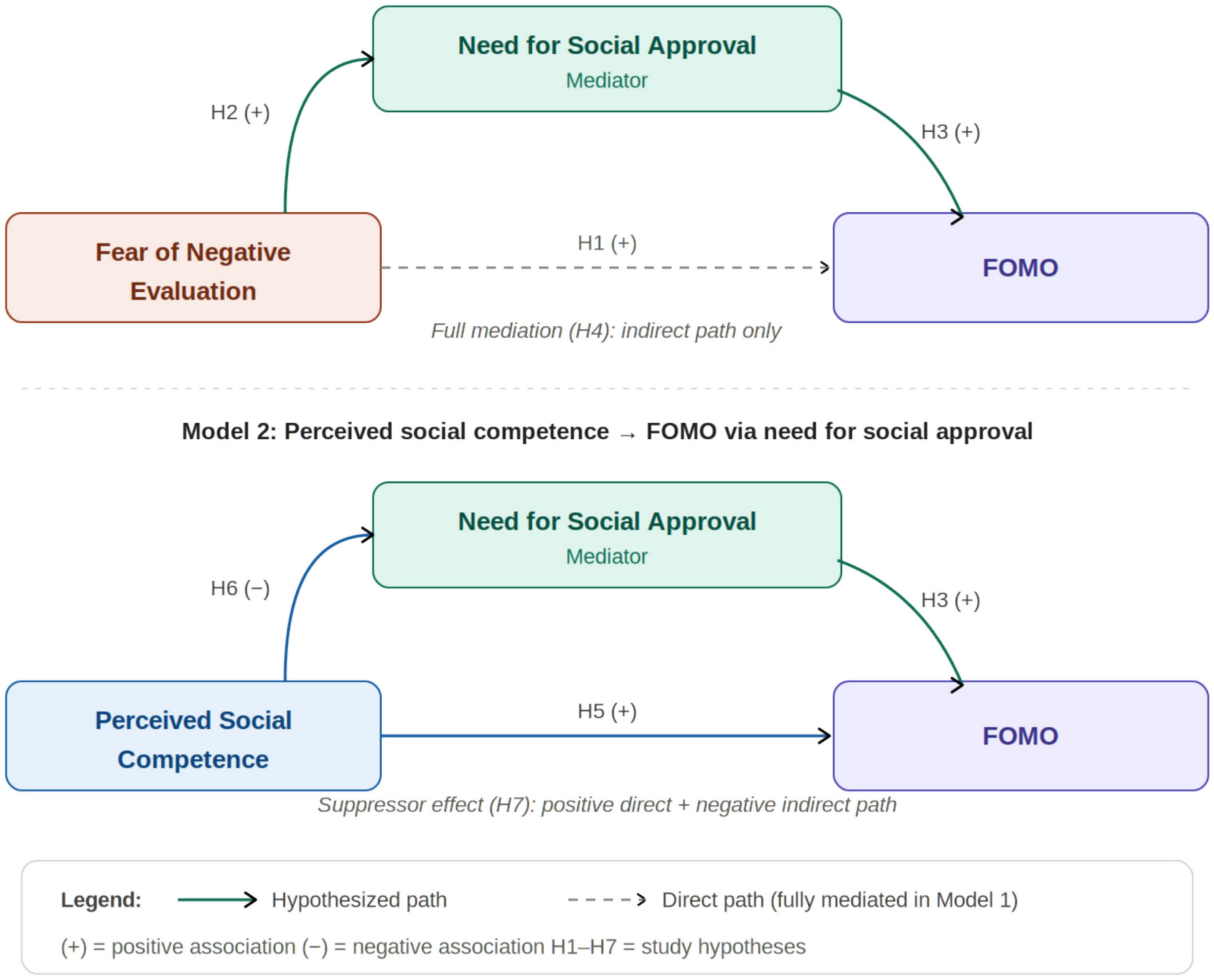
Hypothesized mediation models.

Consistent with prior literature, positive bivariate associations were expected between FNE and FOMO (H1), between FNE and need for social approval (H2), and between need for social approval and FOMO (H3). A negative association was expected between perceived social competence and need for social approval (H6). A positive association between perceived social competence and FOMO was hypothesized based on the exposure-based reasoning outlined above (H5). The central hypotheses concerned the mediating role of need for social approval: H4 predicted that need for social approval would mediate the statistical association between FNE and FOMO, and H7 predicted that need for social approval would mediate the statistical association between perceived social competence and FOMO (see Figure 1).

## Method

### Study design

This study adopted a correlational and explanatory research design within a quantitative paradigm. Correlational designs are particularly suitable for exploring the associations between psychological constructs without manipulating variables (Creswell and Creswell, 2018). More specifically, this study aimed to examine whether the need for social approval statistically accounts for the association between fear of negative evaluation and fear of missing out. A second indirect-association model was also tested, in which perceived social competence was hypothesized to exert a statistical indirect effect on fear of missing out through the need for social approval.

The research was conducted using a cross-sectional design, where data were collected at a single point in time via self-report questionnaires. Cross-sectional approaches are commonly used to examine relational patterns in psychological research (Bryman, 2016), although they inherently limit causal inferences. To test the mediation models, bootstrapped mediation analyses were conducted following the regression-based approach recommended by Preacher and Hayes (2008) and Hayes (2018). This method enables the estimation of indirect effects with increased accuracy and statistical power, particularly for small to medium sample sizes and when the normality of the sampling distribution of indirect effects cannot be assumed. By integrating theory-driven models and empirical analysis, this study aligns with explanatory frameworks aimed at identifying candidate indirect pathways among individual difference variables (Hayes, 2018).

### Participants

A total of 365 participants were included in the study. The sample consisted of emerging adults aged between 18 and 23 years (M = 19.40, SD = 1.25). The majority of participants were female (*n* = 281, 77.0%), while 84 participants were male (23.0%). This gender distribution is consistent with the demographic composition of psychological counseling and education faculty programs at Turkish universities, from which the majority of participants were recruited, and reflects a broader pattern of higher female participation rates in self-report survey research (Rosenthal and Rosnow, 1975). The number of siblings ranged from 0 to 13 (M = 2.75, SD = 1.91), reflecting a diverse range of family sizes. The sample was recruited using convenience sampling, primarily from undergraduate university students in Türkiye. Participation was voluntary, and informed consent was obtained from all individuals prior to data collection. There were no missing data in the dataset, as the online survey was designed in a way that required participants to respond to all items before proceeding to the next section. The adequacy of the sample size for the planned indirect-association analyses was evaluated using guidelines provided by Fritz and MacKinnon (2007), who determined minimum sample sizes required to detect indirect effects at various effect sizes with 80% power using bootstrapping procedures. For medium indirect effects, a minimum of *N* = 88 is recommended; for large indirect effects, a minimum of *N* = 54. The present sample of *N* = 365 substantially exceeds these thresholds, providing adequate statistical power for detecting medium to large indirect effects, consistent with the observed indirect effect in Model 1 (IE = 0.32). For small indirect effects such as that observed in Model 2 (IE = −0.03), Fritz and MacKinnon (2007) recommend a minimum of *N* = 558, a threshold the present sample does not meet. This provides a further empirical basis for the cautious interpretation of the Model 2 indirect effect recommended throughout this paper.

### Data collection tools

#### Fear of missing out scale (FoMOS)

Fear of missing out was measured using the Turkish version of the Fear of Missing Out Scale (FoMOS), developed by Przybylski et al. (2013) and adapted into Turkish by Gökler et al. (2016). The Turkish version of the scale consists of 10 items rated on a 5-point Likert scale ranging from 1 (not at all true of me) to 5 (extremely true of me). Total scores range from 10 to 50, with higher scores indicating greater levels of FoMO. The scale is unidimensional, and no cutoff score is provided. The Turkish adaptation study confirmed the unidimensional structure of the scale through exploratory factor analysis, explaining 39.4% of the total variance. Although this proportion of explained variance is relatively modest for a unidimensional measure, the scale has been widely used in Turkish samples and has demonstrated adequate psychometric properties across multiple studies (e.g., Kaloeti et al., 2021; Tunc-Aksan and Akbay, 2019). The internal consistency of the original Turkish version was reported as *α* = 0.81, and test–retest reliability was also 0.81 (Gökler et al., 2016). In the present study, the Cronbach’s alpha coefficient was similarly high (α = 0.81), indicating good internal consistency.

#### Fear of negative evaluation scale (FNES)

Fear of negative evaluation was measured using the Brief Fear of Negative Evaluation Scale (BFNE), originally developed by Leary (1983) as a shortened version of the Fear of Negative Evaluation Scale (Watson and Friend, 1969), and later adapted into Turkish by Çetin et al. (2010). The Turkish version of the scale includes 12 items rated on a 5-point Likert scale ranging from 1 (not at all characteristic of me) to 5 (extremely characteristic of me). Higher total scores indicate greater fear of being judged unfavorably by others in social or evaluative contexts. The Turkish adaptation study confirmed the scale’s unidimensional structure and demonstrated strong internal consistency (α = 0.87). The scale has shown significant positive correlations with social anxiety and self-criticism, supporting its construct validity. In the present study, the internal consistency of the scale was excellent, with a Cronbach’s alpha of 0.93.

#### The need for social approval scale (NSAS)

The need for social approval was assessed using the Need for Social Approval Scale (NSAS), originally developed in Turkish by Karaşar and Öğülmüş (2016). The scale is designed to evaluate the extent to which individuals seek approval, avoid disapproval, and attempt to maintain positive impressions in social contexts. It comprises 25 items rated on a 5-point Likert scale ranging from 1 (strongly disagree) to 5 (strongly agree). Total scores range from 25 to 125, with higher scores indicating a stronger need for social approval. The NSAS includes three subdimensions: sensitivity to others’ judgments, social withdrawal, and impression management. The original study reported internal consistency coefficients of 0.83, 0.80, and 0.80 for these subscales, respectively, and 0.90 for the total scale. Test–retest reliability was also high (r = 0.90), and the scale showed strong criterion-related validity with measures of social anxiety and fear of negative evaluation. In the current study, the Cronbach’s alpha coefficient for the overall scale was 0.94, indicating excellent internal consistency within the sample. As the present study used the total NSAS score in all analyses, the three-factor structure was not examined separately. This approach is consistent with prior studies that have treated the NSAS as a unidimensional measure when testing mediation models involving overall approval-seeking motivation (e.g., Mürtezaoğlu and Çikrıkci, 2022).

#### Perceived competence scale for adolescents (PCS-A)

Perceived social competence was assessed using the Social Competence subscale of the Perceived Competence Scale for Adolescents (PCS-A), developed by Özer et al. (2016) for Turkish populations. Although the scale was originally normed on adolescent samples, its use in the present study with emerging adults is theoretically and empirically justifiable for several reasons. Developmentally, emerging adulthood (ages 18–25) is widely recognized as a period of continued identity exploration and peer-oriented social development that shares substantial continuity with adolescence (Arnett, 2000). The social competence domains assessed by the PCS-A—including peer relationship formation, social communication, and group acceptance—are not only relevant but arguably central developmental concerns during the university years, a period characterized by the formation of new peer networks and intense social negotiation in both offline and online environments (Arnett, 2000; Desjarlais and Willoughby, 2010). Empirically, adolescent-normed social competence measures have been used with university student samples in prior Turkish FOMO research without reported psychometric difficulties (Tunc-Aksan and Akbay, 2019). In the present study, the internal consistency of the Social Competence subscale was high (*α* = 0.88), providing empirical support for the scale’s reliability in this age group. We acknowledge, however, that confirmatory factor analysis of the PCS-A structure was not conducted in the current sample, and the absence of structural validity evidence for this population is a limitation. Future studies should validate the factor structure of the PCS-A in emerging adult samples or employ instruments specifically developed for this developmental period.

### Data analysis

Prior to conducting mediation analyses, standard assumptions of regression analysis were assessed using built-in diagnostics in Jamovi and R. Normality of the variables was evaluated through skewness and kurtosis values, all of which fell within the acceptable range of ±2 (George and Mallery, 2010), indicating adequate univariate normality. Linearity and homoscedasticity were inspected via residual plots and scatterplots of standardized predicted values versus residuals. No significant deviations were observed. Multicollinearity was examined using Variance Inflation Factor (VIF) scores and tolerance values, both of which were within acceptable thresholds (VIF < 5; tolerance > 0.20), indicating no multicollinearity among predictors. These diagnostics confirmed that the data met the statistical assumptions required for mediation analysis using bootstrapped regression techniques. All statistical analyses were conducted using Jamovi (version 2.3.28) and the R statistical environment (version 4.x; RStudio version 2025.05.1 + 513 was used as the integrated development environment). Descriptive statistics and Pearson correlation coefficients were computed to examine the relationships among the core study variables: fear of negative evaluation, need for social approval, perceived social competence, and fear of missing out. Two bootstrapped mediation analyses were conducted to test the hypothesized models. The analyses followed the ordinary least squares (OLS) regression-based path modeling approach, utilizing 5,000 bootstrap samples and bias-corrected 95% confidence intervals to test the significance of indirect effects. Two mediation models were tested. In the first model, need for social approval was examined as a mediator between fear of negative evaluation and fear of missing out (FOMO). In the second model, the same mediator was tested in the relationship between perceived social competence and FOMO. Only variables with statistically significant bivariate correlations were included in the mediation analyses to ensure model robustness. Demographic variables (age, gender, and number of siblings) were not included as covariates in the primary mediation models in order to preserve parsimony and because the study’s theoretical framework focuses specifically on the social-cognitive pathways linking evaluative concerns to FOMO. However, supplementary analyses controlling for gender were conducted to verify the robustness of the mediation results.

It should be noted that the mediation analyses conducted in this study estimate statistical indirect effects, and the results should not be interpreted as evidence of causal mediation. The cross-sectional nature of the data does not permit the establishment of temporal ordering among variables, and the sequential ignorability assumption required for causal inference (Imai et al., 2010) cannot be verified. The terms ‘indirect effect’ and ‘direct effect’ are used in their statistical sense throughout this paper.

## Findings

### Preliminary analyses

Before proceeding to the mediation analyses, the discriminant validity of the key predictor constructs was examined. The bivariate correlation between fear of negative evaluation (FNE) and need for social approval (NSAS) was notably high (r = 0.84, *p* < 0.001), indicating that these two constructs share approximately 71% of their variance in this sample. This level of overlap raises a legitimate concern regarding whether the mediation model in which NSAS mediates the effect of FNE on FOMO reflects a genuine psychological process or is, at least in part, an artifact of construct redundancy.

To address this concern, a confirmatory factor analysis (CFA) was conducted comparing a theoretically derived two-factor model (FNE and NSAS as separate latent variables) with a constrained one-factor model in which all items were forced onto a single latent variable. The two-factor model demonstrated acceptable fit to the data, *χ*^2^(593) = 1719, CFI = 0.85, TLI = 0.84, RMSEA = 0.060, SRMR = 0.05. Although the CFI value fell marginally below the conventional 0.90 threshold, this is not uncommon in models with a large number of indicators and is considered acceptable when RMSEA and SRMR values meet recommended criteria (Hu and Bentler, 1999). The one-factor model showed meaningfully poorer fit across multiple indices, *χ*^2^(594) = 1958, CFI = 0.82, TLI = 0.81, RMSEA = 0.070, SRMR = 0.05. A *χ*^2^ difference test confirmed that the two-factor model fit the data significantly better than the one-factor alternative, Δ*χ*^2^(1) = 239, *p* < 0.001. To further evaluate discriminant validity beyond the CFA comparison, the heterotrait-monotrait (HTMT) ratio was computed following Henseler et al. (2015). The HTMT ratio for FNE and NSAS was 0.87, falling below the recommended threshold of 0.90 for constructs that are theoretically expected to be related (Henseler et al., 2015). This result provides support for acceptable discriminant validity between the two constructs, indicating that despite their substantial correlation, FNE and NSAS are not statistically equivalent. The latent factor correlation estimated within the two-factor CFA model was 0.86, consistent with the high observed bivariate correlation and reflecting the close theoretical relationship between the two constructs. Importantly, this value also falls below the 0.90 threshold recommended for discriminant validity. Taken together, the CFA model comparison, HTMT ratio of 0.87, and latent factor correlation of 0.86 consistently indicate that FNE and NSAS are empirically distinguishable as separate latent constructs, while acknowledging that the degree of shared variance remains substantial and is discussed further in the limitations section. Nevertheless, the possibility that the full mediation pattern in Model 1 is partly inflated by shared method variance and construct overlap cannot be entirely ruled out, and this is discussed further in the limitations section.

### Descriptive statistics and correlations

Descriptive statistics and Pearson correlation coefficients for the main study variables are presented in Table 1. The analysis revealed several statistically significant associations among the variables. Perceived social competence showed a small but significant positive correlation with fear of missing out (*r* = 0.13, *p* < 0.05), a small negative correlation with need for social approval (*r* = −0.18, *p* < 0.01), and a small negative correlation with fear of negative evaluation (*r* = −0.19, *p* < 0.05). These findings suggest that while socially competent individuals report slightly lower evaluative fears and approval needs, their FOMO levels are not lower—a pattern that will be explored further in the mediation analysis. Fear of negative evaluation was strongly and positively correlated with need for social approval (*r* = 0.84, *p* < 0.001) and moderately correlated with FOMO (*r* = 0.47, *p* < 0.001), supporting the notion that individuals who are anxious about being negatively judged tend to seek more social validation and are more prone to FOMO experiences. Need for social approval showed a significant positive correlation with FOMO (*r* = 0.52, *p* < 0.001), indicating that those with a high need for external validation are more susceptible to experiencing fear of missing out. All significant correlations were in the expected directions and supported the hypothesized relationships among variables. These findings provided the preliminary basis for subsequent mediation analyses.

**Table 1:** Descriptive statistics and correlation coefficients among fear of missing out, fear of negative evaluation, need for social approval, perceived social competence, and perceived academic competence.

Variables	1	2	3	4	5
1. Fear of missing out	—				
2. Fear of negative evaluation	0.469^**^	—			
3. Need for social approval	0.523^**^	0.844^**^	—		
4. Perceived social competence	0.132^*^	−0.188^*^	−0.182^**^	—	
5. Perceived academic competence	−0.002	−0.087	−0.120^**^	0.407^**^	—
Mean	24.1	31.0	73.1	55.5	55.4
Sd	6.86	8.95	18.0	7.91	10.4

^**^p < 0.01, ^*^p < 0.05.

Perceived academic competence was included in the correlation matrix for descriptive purposes only. It was not entered into any of the mediation models, as the theoretical framework of this study focuses specifically on social-cognitive pathways to FOMO.

### Mediation analyses

To examine the statistical indirect association between fear of negative evaluation and fear of missing out (FOMO), a cross-sectional indirect-association analysis was conducted using the need for social approval as a candidate explanatory variable. The analysis revealed a significant indirect effect of fear of negative evaluation on FOMO via need for social approval (IE = 0.32, 95% CI [0.20, 0.44], *p* < 0.001). In contrast, the direct effect of fear of negative evaluation on FOMO was non-significant (DE = 0.05, 95% CI [−0.08, 0.19], *p* = 0.412), indicating a full statistical indirect association. The total effect was statistically significant (B = 0.37, *p* < 0.001), with approximately 86.2% of the total effect being statistically accounted for by the indirect pathway.

These findings suggest that individuals with a higher fear of being negatively evaluated tend to exhibit an increased need for social approval, which in turn was statistically associated with heightened FOMO experiences. The absence of a significant direct path supports the conclusion that the statistical association between evaluative fears and FOMO is fully accounted for by need for social approval, highlighting the statistical relevance of approval-seeking tendencies in this associational pattern. However, given the high correlation between FNE and NSAS (*r* = 0.84), the full statistical indirect association should be interpreted alongside the discriminant validity results reported above. The possibility that the proportion mediated is partly inflated by construct overlap is acknowledged (see Table 2).

**Table 2:** Bootstrapped mediation analysis for need for social approval as a mediator between fear of negative evaluation and FOMO.

Effect type	Estimate	95% CI lower	95% CI upper	*p*
Indirect (IE)	0.32	0.20	0.44	<0.001
Direct (DE)	0.05	−0.08	0.19	0.412
Total	0.37	0.30	0.44	<0.001
Proportion mediated	0.86	0.54	1.21	<0.001

CI, confidence interval, IE, indirect effect, DE, direct effect, Significance based on 5,000 bootstrap resamples using the bias-corrected confidence intervals.

A second indirect-association analysis examined whether need for social approval statistically accounts for the relationship between perceived social competence and fear of missing out (FOMO). The results revealed a small indirect effect in the negative direction (IE = −0.03, 95% CI [−0.07, −0.00], *p* = 0.024). However, the direct effect of social competence on FOMO remained positive and significant (DE = 0.20, 95% CI [0.12, 0.28], *p* < 0.001), suggesting that the overall association was primarily driven by the direct path (Total effect = 0.16, *p* < 0.001). The proportion mediated was negative (−20.6, 95% CI [−0.66, −0.02]), indicating a small indirect effect in the opposite direction of the direct effect rather than classical mediation. Given the marginal magnitude of the indirect effect and the instability of the proportion-mediated estimate, this pattern should be interpreted with considerable caution.

These findings imply that although social competence is generally associated with higher FOMO levels, the need for social approval may act in a complex or even counteracting manner, slightly dampening this effect. The negative direction of the indirect effect suggests that individuals who perceive themselves as socially competent may have a reduced need for approval, which in turn could decrease FOMO tendencies—thereby partially offsetting the direct link. However, given the small effect size and atypical mediation pattern, this model should be interpreted with caution (see Table 3).

**Table 3:** Bootstrapped mediation analysis for need for social approval as a mediator between perceived social competence and FOMO.

Effect type	Estimate	95% CI lower	95% CI upper	*p*
Indirect (IE)	−0.03	−0.07	−0.00	0.02
Direct (DE)	0.20	0.12	0.28	<0.001
Total	0.16	0.08	0.25	<0.001
Proportion mediated	−0.21	−0.66	−0.02	0.02

CI, confidence interval, IE, indirect effect, DE, direct effect, Significance based on 5,000 bootstrap resamples using the bias-corrected confidence intervals.

Collectively, the findings from both indirect-association models offer a nuanced understanding of how social-cognitive constructs are statistically linked to individuals’ vulnerability to FOMO. While fear of negative evaluation appears to be statistically associated with FOMO primarily through a heightened need for social approval, the role of perceived social competence is more complex, with both direct and indirect effects emerging in opposite directions. These patterns underscore the statistical relevance of approval-based motivations in FOMO experiences, and suggest that interventions targeting evaluative concerns and approval-seeking behaviors may hold promise in mitigating FOMO among young adults. These findings are further elaborated and contextualized in the discussion section.

## Discussion

The present study set out to address a specific gap in the FOMO literature: while evaluative fears and approval-seeking motivations have each been independently linked to FOMO, the mechanism connecting them had not been directly modeled. By testing need for social approval as a mediator between FNE and FOMO, and simultaneously examining perceived social competence through the same mediational framework, the study provides two findings that extend prior work in meaningful ways. The primary finding is that the statistical association between FNE and FOMO was fully accounted for by need for social approval, with no significant direct effect remaining. This suggests that evaluative anxiety among emerging adults is not independently associated with FOMO but is statistically linked to it through the motivational pursuit of external validation—a pathway that has been theoretically proposed but not previously tested as a direct model. It should be noted that the present study did not include measures of social exclusion, loneliness, belongingness, or rejection sensitivity. The findings therefore speak to the statistical contribution of approval-based processes and do not establish that these mechanisms operate independently of or in competition with exclusion-based ones. Empirical support for a direct FNE–FOMO association has been limited in the literature (Alabri, 2022; Trepte et al., 2015), though FNE has been shown to positively predict social media addiction (Piko et al., 2024) and to play a mediating role in social media behaviors more broadly (Feng et al., 2025), suggesting that evaluative concerns are implicated in digital social behavior through motivational pathways rather than directly.

A significant positive relationship was found between fear of negative evaluation and the need for social approval. There are similar studies supporting the H2 hypothesis. Zhu et al. (2024) conducted a network analysis of internet addiction, interpersonal sensitivity, FOMO, and online social anxiety among Chinese university students. As a result, evaluative anxiety and FOMO played a significant role between internet addiction and interpersonal sensitivity among university students. Furthermore, evaluative anxiety and the need for approval were reported as the strongest relationship in the social network. Yoshizawa (2020) examined the relationships between approval motivation (praise-seeking and rejection-avoidance) and fear of positive and negative evaluation. In particular, they found that rejection-avoidance-based approval motivation was positively related to fear of negative evaluation. Taken together, these findings support the view that sensitivity to others’ evaluations and the pursuit of social approval are motivationally linked constructs that jointly shape FOMO vulnerability (Yoshizawa, 2020; Zhu et al., 2024).

Another hypothesis, H3, posited that there is a significant relationship between the need for social approval and FOMO. There are studies in the literature supporting this finding. Liang (2017) found significant positive relationships among the need for approval, FOMO, depression, and social media addiction. It has been reported that FOMO is related to being popular and receiving social acceptance (Tanrıkulu and Mouratidis, 2023), and that the needs for belonging and social acceptance are important psychological determinants of FOMO (Li and Liu, 2023). In this study, we found a result indicating that the need for social approval among emerging adults was statistically associated with higher FOMO. It is worth noting, however, that Tandon et al. (2021) found evidence for the reverse direction—that FOMO may intensify approval needs—suggesting that the relationship between these constructs may be bidirectional. This bidirectionality is an important interpretive constraint for the present cross-sectional findings, as the data cannot establish whether approval needs precede FOMO or vice versa.

Wolniewicz et al. (2018) found in their study that FOMO was related to fear of negative evaluation and that FOMO assumed a mediating role in the relationship between fear of negative evaluation and problematic smartphone use. Similarly, FOMO has been found to play a mediating role in the relationship between anxiety and depression and social networking site use (Oberst et al., 2017). These findings in the literature support H4. In emerging adulthood, establishing connections with peers and being accepted by them is very important (Desjarlais and Willoughby, 2010; Kaloeti et al., 2021). Overall, the findings suggest that FOMO is statistically associated not only with social attachment but also with how individuals experience evaluative anxieties and approval-based motivations.

Regarding Hypothesis 5, which pertains to our other indirect-association model, a significant positive bivariate association was found between perceived social competence and FOMO. H5 was formulated at the bivariate level; after accounting for need for social approval in the model, perceived social competence retained a significant positive direct association with FOMO (DE = 0.20, *p* < 0.001), indicating that this relationship holds both as a simple correlation and as a direct path within the statistical model. Deniz (2021) found in a study with university students that social self-efficacy had a significant relationship with FOMO and that FOMO played a mediating role between social self-efficacy and life satisfaction. In another study, FOMO was found to be a significant predictor of social media addiction, and perceived social and academic competence levels were found to be related to these psychosocial processes (Tunc-Aksan and Akbay, 2019). FOMO and social media addiction predict the social competencies of adolescents, and as FOMO and social media addiction increase, adolescents’ social competencies decrease (Ganguly et al., 2022). With a similar result, a negative relationship was found between self-efficacy and FOMO (Lee et al., 2020; Erdoğan and Şanlı, 2019). These findings in the literature differ from the results of our study, and the positive association observed here should be interpreted in light of the exposure-based reasoning outlined in the Introduction rather than as a settled finding.

In Hypothesis 6, a low-level significant negative relationship was found between perceived social competence and the need for social approval. Mürtezaoğlu and Çikrıkci (2022) found in their study with high school students that there was a significant negative relationship between social and emotional self-efficacy and the need for social approval. It is stated that as students’ social and emotional self-efficacy increases, their need for social approval decreases. Karakaya and Ünal (2023) revealed that the need for social approval played a moderating role in the negative relationship between academic self-handicapping and self-efficacy under positive feedback and control conditions.

In the other indirect-association model, while perceived social competence showed a positive direct association with FOMO, it exhibited a small indirect effect in the opposite direction through the need for social approval. Individuals who perceive themselves as socially competent generally appear more prone to FOMO. However, their being less dependent on others’ approval partially reduces this vulnerability. This nuanced structure suggests that social competence may have a dual function. While increased social participation and awareness of social opportunities may elevate FOMO, a reduced need for external approval may limit the intensity of this effect. These results regarding the H7 hypothesis are consistent with recent studies showing that, depending on the regulatory dynamics of socially oriented motivations, FOMO can both strengthen and weaken its relationships with well-being and problematic technology use (Tufan et al., 2025; Groenestein et al., 2024; Servidio et al., 2024; Fioravanti et al., 2021). The Model 2 pattern is more accurately characterized as inconsistent mediation rather than classical mediation. In inconsistent mediation, the indirect and direct effects operate in opposite directions. The inconsistent mediation pattern should therefore be treated as a preliminary observation warranting replication rather than as an established psychological mechanism.

The pattern of opposing direct and indirect effects observed in Model 2 warrants further consideration, though the marginal magnitude of the indirect effect (IE = −0.03, 95% CI [−0.07, −0.00]) and the instability of the proportion-mediated estimate (−0.21, 95% CI [−0.66, −0.02]) caution against strong theoretical conclusions. The positive direct effect of perceived social competence on FOMO (DE = 0.20) is consistent with the possibility that socially competent individuals, by virtue of broader social engagement, may experience greater exposure to social comparison cues and information about others’ activities. One possible explanation for this pattern is the ‘rich-get-richer’ hypothesis (Desjarlais and Willoughby, 2010), which posits that socially skilled individuals leverage digital tools to extend their already active social lives, thereby increasing sensitivity to missed opportunities. However, since social engagement, network size, and social exposure were not directly measured in the present study, this interpretation remains speculative and should be treated as one candidate explanation rather than an established account. Simultaneously, the small negative indirect effect through reduced approval needs is consistent with the possibility that social competence serves a partial protective function by lowering dependence on external validation, though the marginal size of this effect limits confidence in its practical significance.

From an applied perspective, the finding that approval needs statistically account for the FNE–FOMO association suggests that interventions should target approval-based motivational processes rather than FOMO behaviors directly. Cognitive-behavioral approaches that help emerging adults develop self-worth that is less contingent on external validation (Crocker and Wolfe, 2001) may be more effective than strategies focused solely on reducing social media use or digital exposure. Furthermore, the pattern in Model 2 suggests that enhancing social competence alone may paradoxically increase FOMO if not accompanied by efforts to reduce dependence on external approval.

### Limitations and future directions

Several limitations should be acknowledged when interpreting these findings.

The most consequential limitation of the present study concerns the high bivariate correlation between fear of negative evaluation and need for social approval (*r* = 0.84, shared variance ≈ 71%). To evaluate whether this overlap undermines the distinctiveness of the two constructs, two supplementary analyses were conducted. First, a confirmatory factor analysis demonstrated that a two-factor model fit the data significantly better than a one-factor solution (Δ*χ*^2^(1) = 239, *p* < 0.001), indicating that the constructs are empirically distinguishable as separate latent variables. Second, the heterotrait-monotrait (HTMT) ratio was computed following Henseler et al. (2015) and yielded a value of 0.87, which falls below the recommended threshold of 0.90 for theoretically related constructs, supporting acceptable discriminant validity. Nevertheless, the degree of shared variance remains substantial, and the full mediation pattern in Model 1—and particularly the 86.2% proportion mediated—should be interpreted as a statistical description of variance partitioning rather than as evidence of a fully separable psychological mechanism. Future studies should employ behavioral indicators of approval-seeking alongside self-report measures and use latent variable modeling with HTMT-based discriminant validity testing to more cleanly separate these constructs before testing mediation pathways.

Second, although the indirect-association models tested in this study are theoretically meaningful and statistically consistent, causal inferences should be drawn cautiously due to the cross-sectional nature of the research, the collection of data through convenience sampling, and self-reporting from a single source. Additionally, while the sample size of *N* = 365 provides adequate statistical power for detecting medium to large indirect effects (Fritz and MacKinnon, 2007), it falls below the recommended threshold of *N* = 558 for reliably detecting small indirect effects. This represents an additional reason for treating the Model 2 indirect effect with caution, as it may reflect limited power rather than a stable psychological pattern. A further limitation concerns the gender composition of the sample, which was predominantly female (77.0%). This distribution reflects the demographic reality of psychological counseling and education faculty programs at Turkish universities, where female students are consistently overrepresented, as well as the well-documented tendency for women to participate at higher rates in voluntary self-report survey research (Rosenthal and Rosnow, 1975). Nevertheless, the skewed gender ratio constitutes a meaningful limitation for the generalizability of the findings. Prior research has documented gender differences in social anxiety, fear of negative evaluation, approval-seeking behavior, and FOMO (Oberst et al., 2017), raising the possibility that the mediation pathways identified in the present study may not operate equivalently across genders. Although supplementary analyses controlling for gender did not produce substantive changes in the mediation results, the unbalanced gender ratio precludes formal multi-group moderation analyses, which would require more balanced subsamples to yield stable estimates. Future studies should recruit gender-balanced samples and explicitly test whether the proposed pathways from FNE and perceived social competence to FOMO via need for social approval are moderated by gender. It is recommended that future studies be conducted with different samples across different cultures. Longitudinal and experimental studies that investigate in depth the reasons for the emergence of FOMO in individuals will reveal the structure of FOMO. Furthermore, FOMO is a multidimensional phenomenon, and more comprehensive models can be tested with other psychosocial variables. Future studies should examine whether specific NSAS subscales—sensitivity to others’ judgments, social withdrawal, and impression management—differentially account for the indirect pathway from FNE to FOMO. Fourth, the use of an adolescent-normed measure (PCS-A; Özer et al., 2016) in an emerging adult sample represents a methodological limitation, despite the theoretical continuity between adolescence and emerging adulthood with respect to social developmental tasks (Arnett, 2000) and the high internal consistency observed in the present sample (*α* = 0.88). The absence of confirmatory factor analysis for the PCS-A in a university-aged population means that structural validity cannot be assumed, and findings involving this measure should be interpreted with this caveat in mind. Future studies should either validate the PCS-A factor structure in emerging adult samples or use instruments specifically developed and normed for this population.

A related psychometric limitation concerns the CFA model fit obtained in the discriminant validity analysis. The two-factor model yielded a CFI of 0.85, which falls marginally below the conventional threshold of 0.90 (Hu and Bentler, 1999). Although this level of fit is not uncommon in models with a large number of indicators, and the RMSEA (0.060) and SRMR (0.05) values met recommended criteria, the suboptimal CFI indicates that the two-factor measurement model does not fit the data perfectly. This means that the CFA-based evidence for discriminant validity between FNE and NSAS, while supportive, should be interpreted with some caution. Future studies should aim for more parsimonious measurement models, potentially using item parceling or shorter scale versions, to obtain cleaner model fit when testing the distinctiveness of these constructs.

## Conclusion

This study examined two statistical pathways to FOMO among emerging adults, linked by a common candidate explanatory variable: the need for social approval. The primary finding—that fear of negative evaluation was statistically associated with FOMO entirely through approval-seeking tendencies—suggests that approval-based motivational processes represent an additional and statistically proximal pathway to FOMO, complementing rather than replacing exclusion-based accounts. The secondary finding—a small indirect effect in the opposite direction of the direct effect for perceived social competence—underscores the dual-edged nature of social skills in digital contexts. Together, these findings call for a broader conceptualization of FOMO within social-cognitive frameworks and suggest that intervention efforts should target approval-based motivational processes alongside other established mechanisms. However, the interpretive strength of the primary indirect-association finding is tempered by the high construct overlap between FNE and NSAS, and replication with improved discriminant validity evidence is warranted.

## Data Availability

The datasets presented in this article are not readily available because they contain information that could compromise the privacy of research participants. Requests to access the datasets should be directed to Ali Ammar Kurt, aliammarkurt@mersin.edu.tr.
